# Green urine

**DOI:** 10.1002/ccr3.891

**Published:** 2017-03-08

**Authors:** Abhilash Koratala, Muhannad Leghrouz

**Affiliations:** ^1^ Division of Nephrology, Hypertension and Renal TransplantationUniversity of FloridaGainesvilleFloridaUSA

**Keywords:** Green urine, methylene blue, propofol, pseudomonas

## Abstract

Methylene blue is used to assess the integrity of the bowel and may cause self‐limiting bluish or greenish hue to the urine. Green urine is also caused by medications such as propofol and infections such as pseudomonas. Knowledge of the benign nature of this condition prevents unnecessary consultations and anxiety.

## Case Description

**Figure 1:**
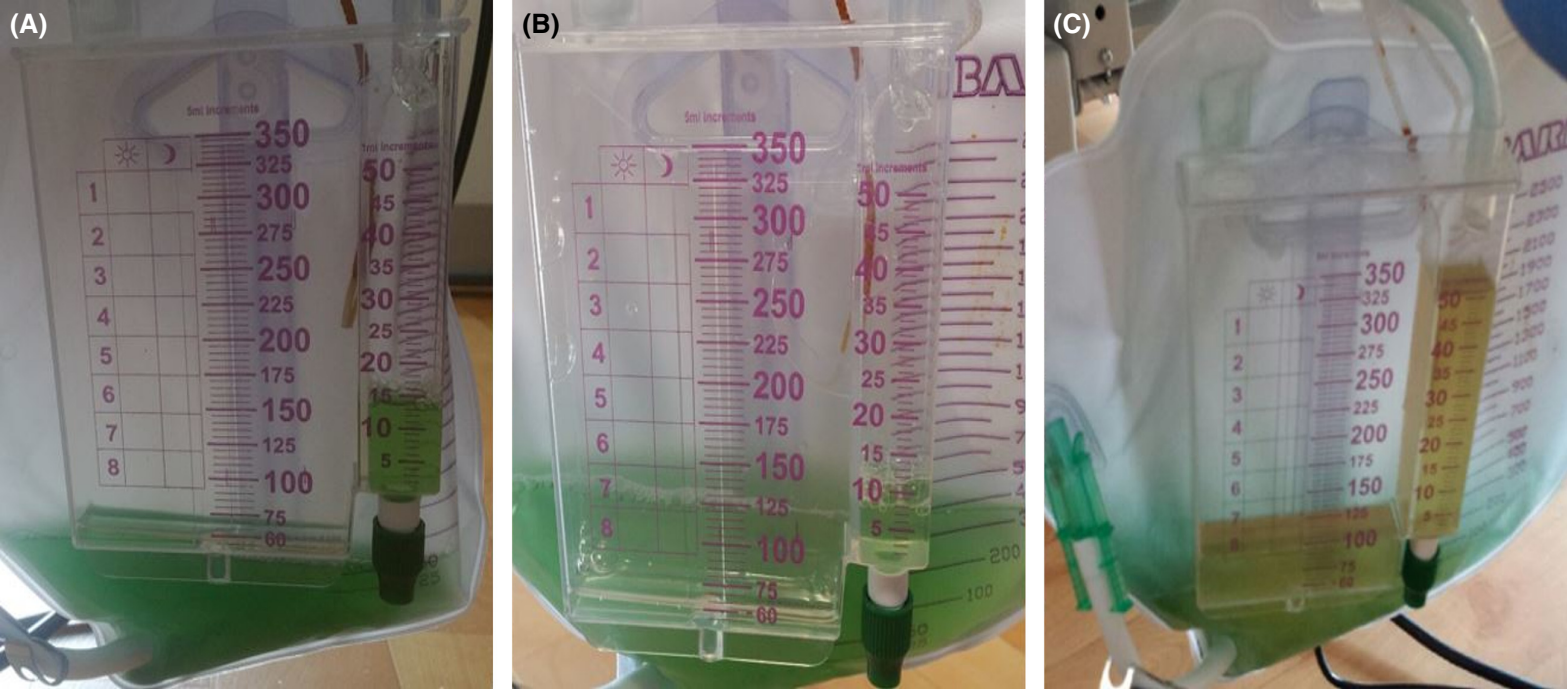
(A) Green urine on the day of methylene blue administration (B and C) fading color of the urine over next few days.

A 62‐year‐old woman was admitted to the hospital for perforated gastric ulcer and underwent emergent exploratory laparotomy with repair of the perforation. She was intubated and sedated using fentanyl. During follow‐up evaluation, she received methylene blue through nasogastric tube to test for integrity of the gastric wall. Subsequently, her urine was noted to be green (Fig. 1A), which gradually faded over a period of 5 days (Fig. 1B and C). Methylene blue is a water‐soluble dye commonly used as a diagnostic aid to assess integrity of the bowel or used as a therapeutic agent in conditions such as ifosfamide‐induced encephalopathy 1 and methemoglobinemia. It is filtered by the kidneys and has no pathologic effects but may cause the urine to have a bluish or greenish hue that is self‐limiting 2. Other known causes of green urine include indigo, *Pseudomonas* infection, drugs such as propofol, cimetidine, amitriptyline, promethazine, and indomethacin.

## Conflict of Interest

The authors have declared that no conflict of interest exists.

## Authorship

Both the authors made substantial contribution to the preparation of this manuscript and approved the final version for submission. AK: drafted the manuscript. ML: acquired the images and other pertinent patient data.
